# Evaluation of Central Hemodynamics in the Diagnosis and Treatment of Hypertension

**DOI:** 10.2174/1573403X19666230511145035

**Published:** 2023-10-02

**Authors:** Pedro Forcada, Jose F. Vilela-Martin

**Affiliations:** 1 Department of Cardiology, Universidad Austral, Pilar, Argentina;; 2 University of Buenos Aires, Buenos Aires, Argentina;; 3 Adjunct Professor at the State Medical School in Sao Jose do Rio Preto (FAMERP), Sao Paulo,Brazil

**Keywords:** Central aortic pressure, arterial stiffness, pulse wave velocity, augmentation index, amplification index, hypertension

## 1 INTRODUCTION

High blood pressure is one of the leading causes of disability and death in developed and developing countries and is the main risk factor for most cardiovascular (CV) complications, like myocardial infarction, stroke, vascular disease, and heart and renal failure. The diagnosis of hypertension seems to be simple, and several guidelines have been written worldwide during the last 30 years to make it easy to diagnose, establish the cardiovascular risk, and determine the treatment. However, hypertension remains largely underdiagnosed and undertreated worldwide, especially in developing countries, which is the main CV risk factor related to major CV complications, and even when it is apparently controlled, a large proportion of patients suffer from complications and have a high CV risk, which is considered a residual risk. Hence, a call to action is urgently needed to modify this situation [1].

Apart from the developed countries, the situation known as “the rule of halves” (half of the patients know they are hypertensives, half of them are not treated, and half of the least is only with blood pressure on target) reveals a lack of awareness regarding hypertension as a cardiovascular risk factor and its adequate treatment and control, with subsequent higher rates of morbidity and mortality, ranking first among the chronic non-transmittable diseases [1].

Although the diagnosis of hypertension seems to depend on blood pressure measurement, its diagnosis and treatment are the most difficult, as mortality seems to be rather more dependent on artery atherosclerosis than on blood pressure levels.

Since 2007, in the European guidelines for the evaluation of the arteries as a target organ and cardiovascular risk marker in global CV risk calculation, further investigation revealed that two-thirds of patients clinically have a lower risk than the level determined by their arterial damage [2].

The level of atherosclerotic disease can be determined using the endothelial function, and in the earlier stage, through the arterial stiffening, the arterial remodeling, and the presence of atherosclerotic plaques, even though these have not been reported to be hemodynamically significant [3, 4].

Arterial stiffening seems to be one of the most attractive targets when detecting early stages of atherosclerotic disease, as it is half a way through the early evanescent endothelial dysfunction and the overt presence of arterial disease-like remodeling and plaques.

## 2 THE ROLE OF CENTRAL HEMODYNAMICS IN THE DEVELOPMENT OF CARDIOVASCULAR DISEASE

The non-invasive blood pressure (BP) measurement represents an excellent predictor of cardiovascular morbidity and mortality. However, the risk reduction in the treatment of hypertension cannot be explained only by the reduction in peripheral BP. From there arises the questioning of the effect of antihypertensive treatment, in addition to the reduction in BP per se, attributing possible effects of the medication on vascular properties independent of the BP effect. Structural and functional vascular changes cannot be fully observed by peripheral BP measurements. Thus, the assessment of BP in other segments of the arterial tree would provide a better visualization of the benefit of treatment on vascular alterations. BP curves differ greatly between the peripheral and central points of the arterial tree (aortic), such that systolic blood pressure (SBP) is much higher in the brachial artery than in the central arteries, while diastolic pressure (DBP) and mean arterial pressure (MAP) differ only slightly [4, 5]. Therefore, central BP is generally defined as BP in the ascending aorta. An increase in central SBP would directly augment left ventricular afterload, causing significant left ventricular hypertrophy and its eventual consequences, such as heart failure and myocardial ischemia. In healthy young subjects, aortic (central) SBP is about 20 mmHg lower than brachial (peripheral) SBP, while MAP and DBP remain stable along the arterial tree. This effect is known as the amplification of SBP or pulse pressure (PP). In older individuals (> 60 years), PP amplification is reduced as a result of greater arterial stiffness and early return of reflection waves so that central BP is practically equal to peripheral SBP, i.e., it is higher than the central SBP of the youngest individuals. Therefore, the relative time interval of the pressure curves, which are transmitted anterogradely and retrogradely in the aorta, is considered an important parameter that defines central BP. Thus, an increase in central SBP caused by retrograde reflection waves, which can be obtained as an index derived from the analysis of the central BP curve of the aorta (augmentation index), depends on the magnitude and time of the waves reflected and indirectly from heart rate and arterial stiffness [4,5]. Consequently, the analysis of central BP could provide an assessment of the differential effect of antihypertensive drugs on the arterial tree since their action is different in vascular structural and functional properties and central hemodynamics [4]. The prognostic reference value of central hemodynamics was clinically demonstrated by the CAFE study (Conduit Artery Function Evaluation Study), a substudy of the Anglo-Scandinavian Cardiac Outcomes Trial (ASCOT), which evaluated the impact of two different BP lowering regimens (atenolol/thiazide-based versus amlodipine/perindopril-based therapy) on derived central aortic pressures and hemodynamics [6,7]. It was found that a greater reduction in central BP compared to peripheral BP using the amlodipine/perindopril combination resulted in a greater reduction in cardiovascular events [6]. A recent systematic review and meta-analysis of randomized controlled trials with 2,498 individuals reported that inhibitors of the renin-angiotensin system and calcium channel blockers are significantly more efficacious than older anti-hypertensive drugs in their effects on central hemodynamics [8].

Central hemodynamics by means of pulse wave velocity (PWV) in several territories, central aortic pressure, and the augmentation and amplification indexes offer several non-invasive, easy and accurate ways to evaluate arterial stiffening, closely linked to the beginning of hypertension and its evolution [4, 5, 9].

Arterial stiffening is different according to age, and the patterns of central hemodynamics are quite different when comparing an adolescent with spurious systolic hypertension, a young male adult, a young premenopausal woman, a postmenopausal woman or the elderly [10, 11].

The precise diagnosis of hypertension, defining the level of arterial disease and the refined decision-making of therapeutics according to these findings as well as the global CV risk, can be achieved using central hemodynamics far beyond what can be estimated by clinical charts, including only clinical and biochemical determinations.

In the 1950s, the arterial stiffness was first determined using formulas related to the PWV with the arterial distensibility by Moens-Korteweg and Braunwell-Hill, which were applied in the 1980s to measure aortic stiffness using the carotid-femoral pulse wave velocity. During the 1990s, a large body of evidence was gathered demonstrating that carotid-femoral (aortic) PWV (c-f PWV) was a reliable CV risk marker. At that time, the formulas to calculate the central aortic pressure, known as the transfer function, were developed and validated. In the 21^st^ century, a consensus was released for PWV and central aortic pressure [12-14]. Then, a meta-analysis validated the power of c-f PWV as a CV risk marker with more than 17,000 patients [15].

A European Joint statement of ESC, ARTERY, and AHA recommendations for arterial stiffness measurement by PWV was released in 2015, and finally, all this evidence was tested using the results of the SPRINT Trial and the SPARTE study [16-19].

Carotid femoral PWV is a strong marker and predictor of CV risk, independent of BP and accurately determines early vascular ageing and the effects of treatment [20-22]. In addition, several clinical conditions can cause changes in PWV (Table **1**) [23].

Central aortic pressure remains complex to be measured; it is poorly understood even when suggestive evidence increased from the CAFE and the BP GUIDE studies about its impact on CV risk and treatment as a surrogate marker of arterial stiffness [24, 25].

## CONCLUSION

In the near future, simplified and cheaper measurements of both parameters will be used in clinical practice. Their response to different cardiovascular and non-cardiovascular drugs and the 24-hour monitoring of arterial stiffness markers are now the new challenges for better and more effective cardiovascular prevention, as they determine the root of the problem, thereby providing a more effective treatment option based on the physiopathology of the arterial disease [26].

In hypertension, arterial stiffness evaluation can be particularly useful beyond its prognostic value and for CV risk restratification in three different situations [27]:

1. In young hypertensives to differentiate spurious isolated systolic hypertension from true hypertension.

2. In middle-aged adults, it detects early vascular ageing and allows early intervention and CV risk reduction. An interplay between PWV and augmentation /amplification indexes leads to a better understanding and treatment of vascular disease as the cause of hypertension.

3. In the elderly, PWV explains the higher pulsatility in isolated systolic hypertension and the main cause of target organ damage; it is a prognostic marker and a therapeutic target. Eventually, the SUPERNOVA patients could be a group of interest in the future in understanding vascular protection mechanisms [28, 29].

## Figures and Tables

**Table 1:** Clinical conditions that may present alterations in pulse wave velocity.

Borderline hypertension
White-coat hypertension
Established hypertension
Diabetes type 1 and type 2
Dyslipidemia
Atherosclerosis
Coronary artery disease
Cerebrovascular diseases
Heart failure
Peripheral vascular disease
Renal failure and dialysis

Others: arrhythmias, growth hormone, smoking, thyroid dysfunction, postmenopausal women, hypotension and postural hypotension, homocysteine

## LIST OF ABBREVIATIONS

AHA: American Heart Association
ARTERY: Association for Research into Arterial Structure and Physiology Society
ASCOT: Anglo-Scandinavian Cardiac Outcomes Trial
BP: Blood Pressure
CAFE Study: Conduit Artery Function Evaluation Study
c-f PWV: Carotid-femoral Pulse Wave Velocity
CV: Cardiovascular
DBP: Diastolic Blood Pressure
ESC: European Society of Cardiology
BP GUIDE: Value of Central Blood Pressure for Guiding Management of Hypertension
MAP: Mean Arterial Pressure
PWV: Pulse Wave Velocity
SBP: Systolic Blood Pressure
SPARTE: Strategy for Preventing Cardiovascular and Renal Events Based on Arterial Stiffness
SPRINT: Systolic Blood Pressure Intervention Trial
SUPERNOVA: Supernormal Vascular Aging
